# Study on the path of high-quality development of the construction industry and its applicability

**DOI:** 10.1038/s41598-024-64786-y

**Published:** 2024-06-26

**Authors:** Dong Wang, Xiaoduo Cheng

**Affiliations:** School of Economics and Business Administration, Heilongjiang University, Harbin, 150080 China

**Keywords:** Five development concepts, Enhancement paths, Applicability studies, fsQCA, NCA, Engineering, Environmental economics, Sustainability

## Abstract

Exploring the influencing factors and enhancement paths of high-quality development of the construction industry is crucial for promoting sustainable development of the construction industry. Based on the concepts of “five development”, this paper takes the construction industry data of 29 provinces (autonomous regions and municipalities) in China as a sample, utilizes comprehensively the combination method of NCA and fsQCA to build a high-quality development driving model of the construction industry, and explores the coupling effect of factors, like the level of scientific and technological innovation, structural degree, precast building model, external market vitality, resources, and environment, on the development of the industry, revealing the path of high-quality development of the construction industry and analyze its applicability. These findings demonstrate that: (1) The level of scientific and technological innovation, the degree of structure, and the vitality of the external market are the core conditions, and a single factor does not constitute the necessary conditions for the high-quality development of the construction industry; (2) There are three paths for the high-quality development of the construction industry, among which the number of representative cases of linkage development led by openness innovation coordination is the largest, and has strong applicability; (3) There are two non-high-quality development paths in the construction industry, and there is a non-simple opposition relationship with the three high-quality development paths in the construction industry.

## Introduction

During the Fourteenth Five-Year Plan, China’s economy entered a new phase of rapid development^1^, and the construction industry, as one of the important industries in the national economy, received more attention for its development^2^. According to the Statistics Bureau, the total production value of the construction industry in 2021 has increased by $1.2 trillion compared to the year 2020, amounting to about 7% of the GDP. At the same time, the number of employees reached 52.8294 million, an increase of over 10 million compared to 2012; The population urbanization rate has reached 64.72%, an increase of 11.62% compared to 2012. Therefore, the construction industry has made important contributions to China’s economic growth^3^, social employment, urbanization^4^, and other aspects. However, with a booming construction market, issues such as imbalanced regional development, poor quality of development, and ineffectiveness, came up. These issues are mainly in the form of high energy consumption, high emissions^5^, extensive development, and low labor productivity^6^, which become more and more prominent with time, this problem has become obvious, seriously hampering the progress of high quality in the construction industry. How to further promote the sustainable and healthy development of the construction industry has turned out to be a great challenge and a top priority.

Five development concepts are a profound shift related to China’s overall development at this time and have a remarkable effect in addressing issues such as developmental momentum and disequilibrium^7^. This application in the field of construction is useful in solving the problem of unbalanced regional development in the construction sector. In 2022, China’s Ministry of Housing and Construction officially published a document entitled “14th Five-Year Plan for the Development of the Construction Industry”, which proposed the framework and associated requirements for innovative and low-carbon development, coinciding with the new development concepts.

The construction industry contributes to 40 percent of the carbon emissions^8^. Consequently, reducing carbon emissions, and representing the trend of low carbon, environmental protection, and sustainable development of the construction industry will be conducive to achieving the dual carbon goal. As a result, there has been a great deal of research by academics into the development trend of the construction industry from different perspectives. Some scholars believe that the construction industry exhibits a consistent and positive development trend. For instance, from the point of view of the construction industry business cycle, Sun et al. ^9^ applied the HP filtering method, in combination with the residual method of measuring fluctuations in the macroeconomic cycle, and tested it with both the random-test model and the test model of the autocorrelation coefficient, and concluded that the peak or trough in the business cycle fluctuation of China’s construction industry occurs approximately once every seven years. Yang et al.^10^ from the point of view of the construction industry chain, there is a steady development trend in the development of the construction industry; analyzed the construction industry input–output table and applied the mean propagation length (APL) model to conclude that the construction industry is closely related to the manufacture of non-metallic mineral products, manufacturing of metal and metal products, and other industries, and the development of the construction industry’s industrial chain has a complex upward trend.

As the research continues to deepen, some researchers believe that the development of the construction industry is affected by the green development behavior of construction companies, and their development patterns are not the same. For instance, Li et al.^11^ based on data from construction firms from the China Bureau of Statistics from 2000 to 2020, used an artificial neural network to construct a prediction model of the influencing factors of green development behavior of construction firms and found that the development of the building industry showed an increasing trend over time.

On this basis, in the context of the Five development concepts by taking panel data from the construction industry in 29 provinces (autonomous regions and municipalities) of China in 2020 as the sample cases, combined use of NCA and fsQCA methods for analysis. The purpose of this paper is to explore multiple factors such as the level of scientific and technological innovation, the degree of structuralization, the scale of prefabricated buildings, the vitality of the external market, and the resources and environment to generate differentiated configuration effects from the development of the building industry, to identify pathways and key influences for the rapid realization of high-quality development in the construction sector. On the other hand, this paper primarily resolves the following questions: (1) What factors make up the core conditions for high-quality development in the construction industry? (2)What are the differentiated pathways to high-quality development in the construction sector?

## Literature review

### Study on influencing factors of construction industry development

Currently, influential factors in the development of the construction industry are primarily focused on three aspects: technological innovation, technology, and industry demand. In terms of technological innovation, Previous research on technological innovation has mainly focused on examining economic models and empirical evidence, such as the impact of fiscal decentralization policies, research and development expenditures, and public debt targets on innovation^12^, as well as the impact of green innovation on reducing carbon dioxide emissions^13^. But some scholars have also conducted research from other perspectives, such as Wang and Qi^14^ have comprehensively investigated the input–output relationship of science and technology innovation in the construction industry through the use of cointegration analysis, the Granger causality test, and other methods, and verified that scientific and technological innovation is conducive to the development of the construction industry. Wang et al.^15^ introduced the support factors of green finance, and established a support system for green finance at multiple levels, leading to the conclusion that scientific and technological input is the fundamental factor. From a technology perspective, Li et al.^16^ adopted the LDA(Latent Dirichlet Allocation)topic model and built on DEMATEL-ANP’s comprehensive assessment system for the construction industry, and concluded the significant impact of digital transformation on the building industry. Chen and Ying^17^ applied the method of principal pathway analysis to analyze 587 papers published between 1989 and 2021 and concluded that the technology of artificial intelligence has a great impact on the scientific and technological advancement of the construction industry. Yousif et al.^18^ carried out a comprehensive review of the literature, discussed the potential application of big data in the construction industry, and concluded that big data has the potential to promote the construction industry. In terms of industrial demand, Wang et al.^19^ used structural equation modeling (SEM) to analyze the relationship between the development and transformation of the construction industry and its influential factors and concluded that stimulating internal and external industries as well as industrial demand had a great influence on the development of the building industry.

### Study on sustainable development of the construction industry

Scholars have conducted a great deal of research into the sustainable development of the construction industry, and the results primarily relate to the development evaluation system and the industrialization of construction. Kamaruzzaman et al.^20^ took Malaysia’s construction industry as an example and established an appraisal system with three levels of objectives, standards, and indicators through the analytical hierarchy process(AHP). As research continues to deepen, some researchers have incorporated the entropy method^21^ and principal component analysis^22^ into the index assessment system. Shamseldin^23^ tried to overcome the shortcomings of evaluation methods by incorporating the efficiency index of the building environment into the evaluation system constructing an assessment model based on different theories and combining it with a variety of methods. Regarding building industrialization, Lu et al.^24^and Lekan et al.^25^ believed that the industrialization of buildings can apply industrial production, information technology, and innovative management^26^, which may not only reduce waste emissions but also enhance work efficiency^27^. In the industrial construction chain, the level of cooperation between firms is considered to be the most important factor^28^. The 4.0 Building, 4.0 industry, and BIM will contribute to the sustainable development of the smart city building industry^29^. Furthermore, to foster the sustainable development of the building industry.

### Study on quality development of the construction industry

Prior literature focused primarily on the influencing factors, connotation, and evaluation system of high-quality development. In terms of influencing factors of development, scholars define high-quality development from different perspectives and analyze the relevant factors. The majority of scholars believe that foreign investment^30^, infrastructure^31^, and environmental regulation^32^ are the primary factors that influence high-quality development. In terms of the development of the construction industry, a few scholars have included carbon emission as an unanticipated output in the high-quality development appraisal system of the construction industry and concluded that the carbon emission constraint, government regulation, scientific and technological input into R&D and upgrading of industrial structures are the major factors^33^. High-quality development has the connotation of exploration and research in various areas such as economics^34^, politics^35^, society^36^, etc. while a high-quality evaluation system involves evaluation indicators such as total factor productivity, and an assessment system from multidimensional perspectives such as policy, economics, and culture^37^.

In summary, researchers have conducted extensive research on the developmental trend, influencing factors, and the sustainable development of the construction industry, and discussed high-quality development, which led to successful academic achievements. However, it suffers from the following shortcomings: (1) existing research primarily focuses on the independent effect of a single factor on outcomes at some level, but does not take into account the synergistic effect between the multidirectional factors behind the development of the construction industry, and it is difficult to explain effectively the development trend, the sustainable, high-quality development of the building industry; (2) In practice, the development of the construction industry is the result of different conditions that match and interact with one another. Existing research has not carried out a more in-depth investigation of the complex causal relationship of construction industry development.

## Model design

### Theoretical basis

Initiatively proposed by President Xi Jinping, the five development concepts, or the development concepts of innovation, harmonization, green, openness, and sharing^7^, are to resolve new contradictions and problems in the process of national economic development, and combined with advanced development experience at home and abroad, which with rich connotations have significant effects in solving developmental problems and increasing the momentum of development^38^. In this paper, we combine the five concepts of development to conduct research, which can better address the unbalanced regional development problem of the construction industry. On the other hand, in the context of the concepts, the development of the construction industry is affected by the five developmental dimensions of innovation, coordination, greenness, openness, and sharing.

### Innovative development

Innovative development factors include the level of scientific and technological innovation^39^. Innovation in science and technology can help improve the productivity and competitiveness of construction companies, realizing economic and social benefits, and providing an impetus for the high-quality development of the construction industry. The lack of innovation and the low level of industry and information technology is one of the major reasons for the extensive development and low efficiency of the construction industry in terms of manpower, which not only severely affects the transformation and modernization of the construction industry but also restricts its sustainable development. An important index of the degree of scientific and technological innovation is the rate of technical equipment.

### Coordinated development

Coordinated developmental factors include the degree of structuralization^40^. Structured degree refers to the structural proportion of the output value of state-owned construction enterprises to the total output value of the construction industry as a whole. State-owned construction companies, which take the lead from the “One Belt, One Road” strategy^41^ to international foreign aid projects, are a major component of China’s construction industry, playing a significant and positive role in transforming and upgrading the construction industry as well as responding to the national strategies. On the other hand, as the degree of structure can be effectively reflected by the proportion of the output value of state-owned construction firms to the total output value of its industry, the proportion is taken as a measure of the degree of structuralization.

### Green development

Green development factors include the scale of prefabricated buildings^42^. Prefabricated buildings have high development prospects^43^. The vigorous development and the expansion of the scale of the prefabricated construction industry are conducive to the achievement of the “two-carbon” goal^44^. It is acknowledged that traditional cast-in-place buildings fail to reduce carbon emissions, while prefabricated buildings featured in both low-carbon and environmental protection, can fundamentally address this issue. Given the capability to integrate standardization, technology, and resource base of the prefabricated construction industry, it can drive the rapid development of the prefabricated construction industry chain and achieve scale effects. To measure the scale of the prefabricated building, we take the number of industrial bases of prefabricated buildings as an index.

### Open development

Open development factors include the vibrancy of the external market^45^. High external market vitality is conducive to the development of internal and external linkages in the construction industry and to the improvement of the quality of openness^46^. The construction industry is believed to be one of the first industries open to the outside world in China, so construction companies have a long history and rich experience in exploring overseas markets. Data from the Ministry of Commerce show that China’s newly signed overseas project contracts in 2021 have increased by 1.2% year on year from 2020 to $258.49 billion, and with further promotion of national strategy and an urgent need for business transformation and upgrading, this proportion will continue to rise^47^. The externality, the proportion of the turnover of projects under contract to the output value of the construction sector, can effectively explain the vitality of the external market, and so externality can be used as a measure of the vitality of the external market.

### Shared development

Shared development factors include resources and the environment^48^. By optimizing the resources and environment of the construction industry, the rate of social employment will increase. According to Bureau statistics, the ratio of the construction industry to society as a whole in 2020 is approximately 7.15 percent, with a population of 53.67 million and 750.64 million, respectively. Furthermore, during COVID-19, the government implemented closed management as a result of the spread of the pandemic, leading to the closure of some businesses, and the number of unemployed people in the country grew year-on-year. In the face of such challenges, construction firms are still expanding the scale of recruitment at the social and school levels, and are continuing to recruit professionals which hugely contribute to addressing the problem of difficult employment and to stabilizing the social employment rate. The ratio between the number of people employed in the construction industry and the number of people employed in the whole society can effectively explain the resource and environmental conditions and so we take it as a measurement index of resource and environment.

### Model construction

**Figure 1:**
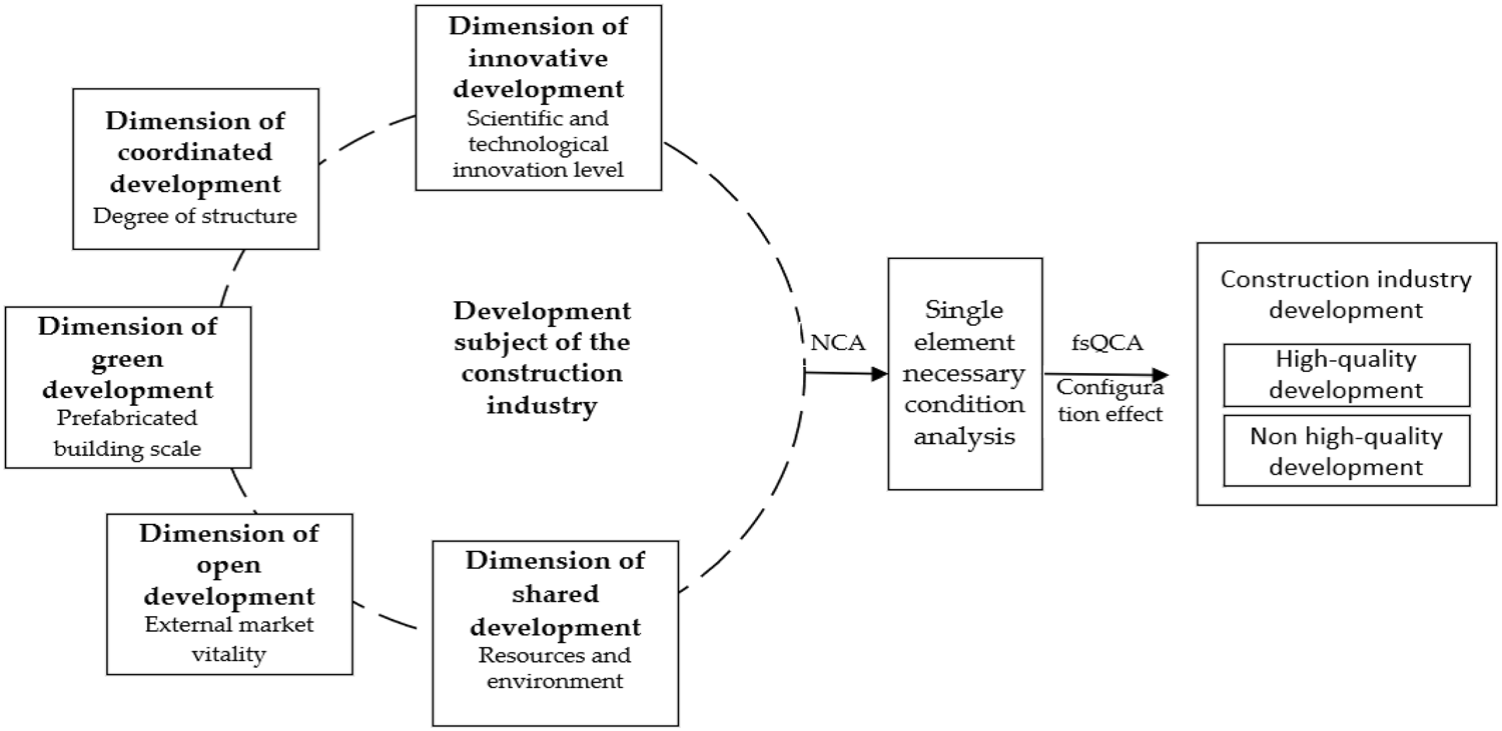
Development driving model of the construction industry.

In summary, under the background of the five development concepts, the following driving model is built by the combination of the NCA and fsQCA (Fig. 1).

## Research methods and data sources

### Combination method of NCA and fsQCA

The fuzzy set qualitative comparison method is a fuzzy set method based on fuzzy mathematics^49^. It can quickly analyze complex systems and social phenomena, explore the essential laws of phenomena, and propose effective solutions. It combines system theory with system analysis and can be described using mathematical models. Its mathematical model includes defining elements, evaluating element types, and describing elements, fully solving linear programming problems in mathematical model formulation. Existing software includes the “MANAGER”, “Simplex method”, and “CAE (Computer Aided Engineering)” developed in the UK. Among them, “MANAGER” is the most commonly used and has a faster calculation speed compared to previous fuzzy set methods. The fsQCA method can optimize the system by using the objective function or the vector of the entire function to solve environmental impact problems.

fsQCA is a case study-oriented research method proposed by Ragin in 1987 and based on Boolean algebraic operation and set theory to explore the complex relationship between antecedent and consequent variables. This method focuses on analyzing configuration effects, in which the cases are equated with the configuration of conditional variables, and the conditions, instead of being independent, are believed to be interdependent. Case analysis helps to identify the causal relationship between the conditional configuration and the outcome, and the conditional configuration inducing the outcome or not is checked^50^. In this paper, we adopt the fsQCA method to explore the path for high-quality development of the construction industry primarily based on the following points: First, distinct from traditional linear analysis, the set-theoretic fsQCA method draws clearer and more precise conclusions. Secondly, fsQCA focuses more on the incentive caused by combining multiple conditions and believes that the condition variable and outcome variable have a non-linear and replaceable relation, which is more suited to exploring the differentiation pathway of multi-factor coupling in the development of the construction industry. Thirdly, different from studying large sample data through traditional methods of analysis, fsQCA has low sample number requirements and is best suited to the study of small and moderate sample sizes.

NCA is a method that identifies and tests the effects of outcome variables on sample data^51^. While the fsQCA method may also identify necessary conditions leading to the results, it focuses more on qualitative research. That is, it is limited to answering only whether the conditions are necessary for the outcomes, but cannot explore the degree of necessity in depth ^52^. NCA, on the other hand, can accommodate both qualitative and quantitative analysis. Not only can it answer whether the condition is necessary for the outcome, but it can also quantitatively analyze its degree of necessity (Please refer to Tables 2 and 3 for details). In the fsQCA case, the NCA method can address its shortcomings^53^. By combining NCA and fsQCA methods, more accurate results can be obtained^54^, which will make the analysis more compelling.

### Data source and variable measurement and calibration

#### Data Source

In this paper, we take 29 provinces (autonomous regions and municipalities) within China as our sample cases, which are outside the scope of this study because of the incompleteness of the data from Hong Kong, Macao, Taiwan, Tibet, and Hainan. Among them, the rate of technical equipment, the number of employees in the construction industry, and the whole society come from the China Statistical Yearbook, the output value data of the construction industry comes from the China Statistical Yearbook of the construction industry, the assembled industrial base data comes from the website of the Ministry of Housing and Construction, and the turnover data of foreign contracted projects comes from the website of the Ministry of Commerce. Using data from the 2020 construction industry as the research base for both NCA and fsQCA, this paper explores the path of high-quality development of the construction industry and the complex mechanisms existing in the development process of the construction industry in different regions.

#### Measurement of variables

##### Result variable

Given the enormous differences in economic scale and the number of employees in various provinces and cities, relative indicators can effectively reduce the incidence of fundamentals, and simultaneously referring to Hua et al.’s research, improvements in production efficiency are the foundation of high-quality development^7^, and labor productivity in the construction industry is set as the outcome variable, and the high-quality development of the construction industry in various provinces and cities is measured by the labor productivity of the construction sector.

##### Condition variables

The level of scientific and technological innovation. Previous research on technological innovation mainly focused on examining economic models and empirical evidence^12^, but neglected the overall innovation level of the industry, and the technology-equipment ratio can to some extent measure the overall level of technological innovation Therefore, this article calculates the level of scientific and technological innovation in the construction industry in each province (region, city) in 2020 through the rate of technological equipment;

Degree of structure. If we calculate the ratio of the output value of the state construction industry to the total value of the output of the construction industry, the degree of structure of the construction industry in each (district and city) in 2020 is measured;

Prefabricated building scale. Prefabricated buildings refer to buildings that are assembled using prefabricated components on construction sites^55^. By counting the number of prefabricated industrial bases, the prefabricated building scale of each province (autonomous region, municipality) in 2020 was calculated.

External market vitality. The foreign market vitality of the construction industry is estimated by calculating the proportion of the turnover of foreign contracted projects in the construction industry output value of each province (autonomous region and municipality) in 2020.

Resources and environment. The resource environment of the construction industry is calculated by calculating the ratio of the number of employees in the construction industry of each province (autonomous region, municipality) to the whole society in 2020.

##### Calibration of variables

To calibrate with the quantiles from the initial data, we used the direct calibration method, and the 95%, 50%, and 5% quantiles correspond to the calibration points of complete membership, crossover, and complete non-membership, respectively^56^. Referring back to the research of Du et al.^57^, the calibration of non-high-quality development in the construction industry is achieved through the non-set of its high-quality development. The relevant descriptive statistics are shown in Table 1.

**Table 1:** Development variable set, calibration, and descriptive statistics of the construction industry.

Set	Fuzzy set calibration			Descriptive statistics	
	Full subordination	Crossing point	Completely unaffiliated	Mean value	Standard deviation
Labor productivity	642,269.200	423,307.001	318,075.800	450,311.172	107,993.705
Scientific and technological innovation level	25070.200	10624.001	4379.800	12913.000	9234.643
Degree of structure	1.214	0.330	0.080	0.454	0.399
Prefabricated building scale	26.600	10.001	1.800	11.414	8.231
External market vitality	8.154	1.951	0.367	2.579	2.351
Resources and environment	0.692	0.168	0.019	0.246	0.257

## Empirical results and analysis

### Analysis of the necessary conditions

Necessity analysis of antecedent conditions is a prerequisite for path analysis^58^. The first is to use the NCA method to test whether a single element is a necessary condition for the high-quality development of the construction industry in varying degrees. The results of the analysis of the utility and bottleneck levels of the NCA method are shown in Tables 2 and 3, respectively. The fsQCA method is then used to further verify the necessary results from the single-element NCA analysis. The results of the necessary condition tests of the fsQCA method are shown in Table 4.

### NCA necessity analysis

#### Utility analyses

The conditional effect sizes were calculated using the CR (Ceiling Regression) estimation method of upper-bound regression and were based on the principle of utility-size analysis. Only when the effect size of a certain factor is higher than 0.1 and the Monte Carlo simulation permutation test result is significant, this factor is seen as a necessary condition^57^. Among them, the analysis results in Table 2 will prove whether the antecedent variable constitutes a necessary condition for the high-quality development of the construction industry. Table 2 shows the effect size (d) of the degree of structuring, the scale of the prefabricated buildings, the vitality of the external market and the resource environment are all less than 0.1, and the *P* value is higher than 0.01; while the effect size (d) of the level of technological innovation is high at 0.1, and the *P* value is higher than 0.01. We can infer that all variables fail to satisfy the conditions that d is greater than 0.1 and *P* is less than 0.01 at the same time, indicating that none of the antecedent variables is a necessary condition for high-quality development in the construction sector.

**Table 2:** Analysis of NCA necessary conditions for the development of the construction industry.

Antecedent condition	Method	Accuracy (%)	Upper limit region	Range	Effect size d	*P*-value
Scientific and technological innovation level	CR	86.2	0.150	0.93	0.161	0.073
Degree of structure	CR	96.6	0.078	0.92	0.085	0.308
Prefabricated building scale	CR	96.6	0.049	0.92	0.053	0.372
External market vitality	CR	93.1	0.083	0.92	0.090	0.315
Resources and environment	CR	89.7	0.037	0.93	0.040	0.395

#### Bottleneck-level analysis

Referring to the research of^59^ Bottleneck Levels (in%) using CR, NN indicates unnecessary, and its meaning is explained using Table 3 as an example to explain the table data (the construction industry reaches a development level of 10%, and the level of scientific and technological innovation is not a necessary condition for the development of the construction industry, which has reached 40%, with a necessary level of 0.8%). Table 3 shows that for the construction industry to reach 100% development, the five antecedent conditions of the level of technological innovation, degree of structuralization, scale of prefabricated buildings, external market vitality, and resources and environment, the levels of need reach 52.9% and 99.9%, 53.5%, 61.1%, and 55.6%, respectively. In terms of the degree of necessity, the bottleneck level of external market structuralization and vitality is relatively high, and the bottleneck level is more than 60% higher than the other conditions.

**Table 3:** NCA bottleneck level analysis of pre-development conditions in the construction industry.

Construction industry development	0	10	20	30	40	50	60	70	80	90	100
Scientific and technological innovation level	NN	NN	NN	NN	0.8	9.5	18.2	26.9	35.5	44.2	52.9
Degree of structure	NN	NN	NN	NN	NN	NN	NN	NN	NN	41.1	99.9
Prefabricated building scale	NN	NN	NN	NN	NN	NN	NN	NN	NN	26.3	53.5
External market vitality	NN	NN	NN	NN	NN	NN	NN	NN	19.8	40.5	61.1
Resources and environment	NN	NN	NN	NN	NN	NN	NN	NN	NN	17.2	55.6

#### fsQCA necessity test of antecedent conditions of construction industry development

To verify the NCA necessity analysis results, the fsQCA method was employed. If the coherence of the condition is greater than 0.9, the condition is taken to be the necessary condition for the result^60^. The fsQCA necessity test results are shown in Table 4, among them ~ indicates a lack of antecedent conditions, such as Scientific and technological innovation Level represents a high level of technological innovation, and ~ Scientific and technological innovation Level is a non-set of levels of technological innovation, which is equivalent to a low level of technological innovation. The results show that the coherence level of all conditions is no greater than 0.9. This provides evidence that a single factor is not a necessary condition for the development of the construction industry, which is the same as the outcome of the analysis of the NCA.

**Table 4:** fsQCA necessity test results.

Condition Variable	High-quality development		Non-high-quality development	
	Consistency	Coverage	Consistency	Coverage
Scientific and technological innovation level	0.701	0.744	0.492	0.537
~ Scientific and technological innovation level	0.564	0.519	0.765	0.725
Degree of structure	0.680	0.740	0.542	0.608
~ Degree of structure	0.640	0.576	0.768	0.712
Prefabricated building scale	0.654	0.704	0.562	0.623
~ Prefabricated building scale	0.649	0.590	0.733	0.686
External market vitality	0.700	0.777	0.526	0.601
~ External market vitality	0.641	0.568	0.805	0.734
Resources and environment	0.526	0.585	0.637	0.729
~ Resources and environment	0.756	0.669	0.638	0.581

#### Configuration analysis of construction industry development conditions

We used the software fsQCA 3.0 to compute the conditional configuration of calibration data from 29 provinces (autonomous regions and municipalities). Referring to existing research^61^, both the coherence threshold and the frequency threshold were fixed at 0.8 and 1, and the PRI(Proportional Reduction in Inconsistency)threshold to 0.7, and finally, the qualitative fuzzy set comparison method was applied to obtain the complex solutions, the intermediate solutions, and the simple solutions. Complex solutions and intermediate solutions yield the same results, intermediate solutions account for both coverage and simplicity, and intermediate solutions are for the most part analyzed by intermediate solutions. Thus, the condition on the kernel that arises simultaneously with the intermediate solution and the parsimony solution is the edge condition^62^, and the result is given in Table 5. The results show that the overall coverage of the development of high/non-high-quality in the construction industry is 0.507 and 0.532, respectively, approximately 50% of the sample can be included in the analysis, and three configurations of high-quality development elements are achieved, namely S1, S2 and S3. NS1 and NS2 are two non-high-quality development factor configurations. On the other hand, it is also found that there is no correspondence between the non-high and high -quality development configurations, and the variable configuration resulting in the non-high/high quality development is not symmetric.

**Table 5:**
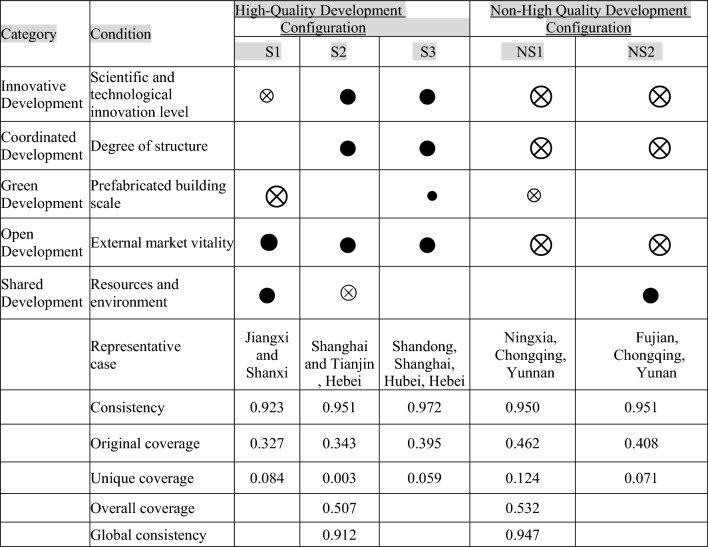
Development Configuration Analysis Results of the Construction Industry (N = 29).

indicate the condition to appear,  indicate the condition does not appear,  indicate the core conditions,  indicate edge conditions. Blank space indicates whether or not the condition is acceptable.

### Analysis of high-quality development pathways and their applicability

#### Configuration S1 is an open shared development path

For the S1 configuration, the coherence and original coverage were 0.923 and 0.327 respectively, and 32.7% of the samples were able to be covered. In this configuration, the construction industry plays a pivotal role in the vibrancy of the external market and the natural resource environment, despite the small scale of the prefabricated building and the low level of science and technology innovation, since it can achieve high-quality development of the construction industry, Therefore, it is named as an open shared development path.

Jiangxi Province represents the S1 configuration. While the number of prefabricated construction industry bases in Jiangxi Province ranks sixteen in the nation, and the construction industry’s technical equipment rate ranks 21st in the nation, the population of Jiangxi Province ranks 13th in the country, and it has many well-known colleges and universities, such as Nanchang University, Jiangxi University of Finance and Economics and many professional colleges like Jiangxi Vocational and Technical College. The enormous number of talent in colleges and universities and the population base of the province provide a huge guarantee for the talent pool of the construction industry, and the number of construction industry employees ranks twelve in the nation. At the same time, Jiangxi Province is deeply integrated into the co-construction of the “Belt and the Road”, which is of great importance to international economic and commercial cooperation, and has a relatively high degree of outward focus in the building industry. The results show that the provinces represented by Jiangxi Province are in line with the open shared development path. The representatives of this path all come from the central and western regions, indicating that this path is not restricted by regions and has a certain degree of applicability. However, the small number of representative cases indicates that its applicability is average.

Given the characteristics of this path, it is more suitable for regions with external market vitality and resource environment as the core and vigorously promotes the high-quality development level of the construction industry. At the same time, it also enlightens that the relevant regions can speed up international economic cooperation, seeking to increase the vitality of the external market and to give the full game to the benefits of the people, higher vocational colleges and universities in the area, and focus on two aspects to achieve develop rapidly.

#### Configuration S2 is an open innovation coordinated development path

For configuration, S2, the coherence, and original coverage are 0.951 and 0.343, respectively, and it is possible to cover 34.3% of the samples. Under this configuration, the level of scientific and technological innovation, the degree of structure, and the vitality of the external market of the construction industry play a central role, which may ignore the size of the prefabricated building, and may achieve the high-quality and rapid development of the building industry even in the absence of resource and environmental benefits. Therefore, it is named as an open innovation coordinated development path.

Tianjin is a typical representative of configuration S2. In the “China Regional Science and Technology Innovation Evaluation Report 2020”, Tianjin was ranked 4th in the overall level of innovation in science and technology, and the construction industry’s technical equipment rate ranked 11th in the country. At the same time, Tianjin is home to numerous state-owned construction companies such as MCC Tiangong Group Corporation Limited and China Construction Sixth Engineering Bureau Corp. LTD which have played an enormous role in the development of the building industry, based on data from the construction industry in the 2020 China Statistical Yearbook, the proportion of state-owned enterprise construction industry output in the city ranks 14th in the country. Moreover, as a major hub of the “Belt and Road”, Tianjin enjoy unparalleled advantages in geography, location, and resources compared to other cities. Statistics show that The city’s ratio of completed turnover of outsourced projects is the first in the country and that the external market is vigorous. The results show that the region represented by Tianjin conforms to the path of open innovation coordinated development. From the representative regions of this path, it only covers provinces and cities in the eastern region, indicating that this path is limited by the region and its applicability is relatively weak. However, the number of representative cases proves that its applicability is good.

Given the characteristics of this path, it is more suitable for regions with a focus on technological innovation level, structural degree, and external market vitality to improve the high-quality development level of the construction industry. These results suggest that relevant regions may place importance on investing and developing scientific and technological innovation, giving full play to the inherent advantages of state enterprises in the region, strengthening international economic cooperation, expanding foreign trade, and achieving high-quality development of the building industry.

#### Configuration S3 is a collaborative development path led by openness, innovation, and coordination

The consistency and original coverage rate of configuration S3 are 0.972 and 0.395, among which, out of the three configurations, this particular one has the highest original coverage rate and can be understood for 39.5% of the samples. In addition, technological innovation level, structural degree, and external market vitality are the core conditions, while the prefabricated building scale is the marginal condition.

Shandong Province is representative of the S3 configuration. In 2020, the regional innovation capability of Shandong Province ranks 6th in the country, and the construction industry’s technical equipment rate is 5th in the nation. Shandong Province is thus shown to have high levels of technological innovation. At the same time, Shandong Province places a high value on the development of the construction industry at the level of industrialization, greening, and intelligence, vigorously promoting and expanding the scale of assembled buildings and the construction of industrial bases. The number of prefabricated building industry bases in the province Ranked first in the country also supports this point from the statistical data. The Shandong Provincial Party Committee and the government also place a high value on the development of state-owned enterprises in the construction industry and emphasize the promotion of strategic cooperation between the provincial government and large-scale state-owned construction enterprises. As a result, the state-owned enterprise has played a significant role in transforming and upgrading the province’s construction industry. According to statistics, the construction industry of state enterprises in the province accounts for 6th of the nation’s total production value, which can also be proven. At the same time, the Shandong provincial government supports construction companies to “go out” to expand their overseas business and increase their international market share. Data from the Ministry of Commerce show that the proportion of the turnover of foreign-contracted projects relative to the total production value of the construction industry in that province ranks among the highest in the country, and supports foreign-invested companies to invest and develop in this province, and enhances its development environment, policies, and systems, showing vibrancy in the foreign market. These results show that the provinces represented by Shandong Province are in line with the linkage development path led by openness innovation coordination. In terms of the representative regions of this path, it covers some provinces and cities in the eastern and central regions and represents the largest number of cases, indicating that this path has strong applicability and degree of applicability.

Given the characteristics of this path, it is more suitable for regions with technological innovation level, structural degree, and prefabricated building scale as the core, and parallel moving prefabricated building scale as the auxiliary to achieve high-quality development of the construction industry.

### Analysis of the non-high quality development path and its applicability

Table 5 shows that there are two configurations for the non-high-quality development of the construction sector. In the NS1 configuration, regardless of the resource environment, low levels of scientific and technological innovation, low degree of structuration, and lack of external market vitality are the fundamental conditions, and the low scale of prefabricated buildings is the marginal condition, which plays a role in the non-high-quality development of the construction industry. Note that the uniformity of this configuration is 0.950, and the original coverage is 0.462, which is higher than the original coverage of the NS2 configuration, which indicates that the NS1 configuration is the primary reason for the non-high-quality development. In the NS2 configuration, even though the region has the advantage of good resources and environment, high-quality development will be limited if the government does not pay attention to improving scientific and technological innovations, ignores the structure of the construction industry, and lacks the vitality of the external market.

### Robustness test

Referring to the research of Zheng et al.^63^ the robustness of the results is verified by changing the consistency threshold and PRI value, that is, the consistency threshold is changed from 0.8 to 0.85, the PRI is changed from 0.7 to 0.75, and the value of 1 is set as the threshold for the frequency of occurrence. The conclusion of the resulting configuration is consistent with the original configuration, proving that the results of this paper are robust.

### Necessity of single condition for high-quality development

Firstly, although some scholars believe that the development of the construction industry is influenced by individual factors such as technological innovation^14,15^ and technological^16,17^ industry demand^20^, the necessity analysis results of applying NCA in this article show that the high-quality development of China’s construction industry is the result of the synergistic effect of multiple factors, and a single condition does not constitute an obstacle to the high-quality development. In the analysis at the bottleneck level of the NCA, it is worth noting that the results show that to reach 90% of the development level of the construction industry, the necessary level of scientific and technological innovation rises to 44.2%, which is higher than the necessary level of the other conditions. This can refer to the broad application of scientific and technological innovation technology in construction, which has had a positive impact on the development of the construction industry such as BIM technology, 3D printing technology, and other information technologies in the promotion and application of the construction industry. However, to achieve 100% of the construction industry’s development level, the required level of external market structuring and vitality rises to 99.9% and 61.1%, respectively, which is higher than the necessary level of the other conditions indicating that the degree of structuring and the vitality of the external market play a leading role in the high-quality development. Shanghai, for example, is one of the cities with a large number and scale of state-owned construction companies, a large proportion of the total value of output comes from the output value of state-owned construction firms, and there is a high degree of structure in the construction industry, which deeply incorporates the “Belt and Road” strategy, is actively exploring overseas engineering markets, has a strong vitality in the overseas market and its construction industry has a high degree of development.

In addition, Th analysis of the bottleneck level of the NCA also shows that the scale of prefabricated buildings and the resource environment also have a certain level of bottleneck, but rather the bottleneck level of scientific and technological innovation, the degree of structuring, and the vitality of the external market in the construction industry development process (90% and 100% development level). These results show that a high level of science and technology innovation, a good degree of structure in the construction industry, and high external market vibrancy are more conducive to high-quality development of the construction industry.

### Specific strategies and intervention measures for enhancing the pathway

The enhancement path of high-quality development in the construction industry requires the formulation of a series of strategies and intervention measures to ensure the steady implementation of the path. Specific strategies include growth-oriented strategies and diversification strategies. The growth strategy refers to utilizing government support, regional advantages, and industrial advantages to actively expand the scale of green buildings represented by prefabricated buildings. However, due to the high cost of promoting green buildings in the early stage and the low investment willingness of construction enterprise developers, the steady promotion of green buildings is affected. This requires the government to formulate specific government subsidies and other measures to intervene and ensure the steady development of green buildings. The diversification strategy includes building regional cooperation, industrial integration, etc., to achieve complementary advantages and resource industry sharing. However, due to the limitations of communication between enterprises, the government must take the lead in intervening and building a green building industry platform and technology exchange platform to ensure precise docking between enterprises. In addition, green buildings have made certain progress in building energy-saving technology, low-carbon material technology, and intelligent energy-saving technology, and have achieved factory production. At the policy level, with strong support from the government in the early stage, green buildings have gradually been recognized and accepted by enterprises and society, and a sound standard system has been further developed. The large-scale development has also reduced construction costs for enterprises, and the government has reduced its intervention in the market. From a practical perspective, the high-quality development path of the construction industry can be guided by policies in the early stage, with improving the level of technological innovation, structural degree, and external market vitality as the core to achieve high-quality development of the construction industry.

### Enhance the practical feasibility and scalability of the path

Firstly, this article analyzes the construction industry in China as a sample, which has certain limitations. However, China’s construction industry market share accounts for 30% of the global market share, and the total output value of the construction industry ranks first in the world, far exceeding other countries. Its high-quality development experience in the construction industry is worth learning and learning from in other regions and countries. Secondly, as a developing country, China has the same or similar background and industrial structure as other developing countries such as India in Asia, Russia in Europe, and Brazil in Latin America. The high-quality development path also has certain applicability. Finally, through the Belt and Road strategy and global economic integration, China’s construction industry has strengthened its close ties with other countries in the world. Its standard system, construction technology, methods, etc. have had a certain influence in cooperative countries. These countries can also learn from China’s high-quality development path and experience.

## Discussion

### Differentiated path selection for high-quality development

This article uses a combination of NCA and fsQCA methods to further enrich the research methodology system, making the research results more fine-grained and robust^58^. Based on the analysis of the development configuration of the construction industry, it is not difficult to find representative cases in the high-quality development path of the construction industry. The eastern region has the most provinces and cities, followed by the central region and the western region, with only Shaanxi Province as the representative case. The representative cases that appear in the non-high-quality development path of the construction industry, except for Fujian, are all provinces and cities in the western region. This indicates that the construction industry as a whole is showing an uneven development trend with high development quality in the eastern and central regions and low development quality in the western regions. It is worth noting that provinces and cities in the eastern region, represented by Fujian, appear in non-high-quality development paths, while provinces and cities in the western region, represented by Shaanxi, appear in high-quality development paths. This also indicates that the development of the construction industry is to some extent influenced by regional and other factors. Economically underdeveloped provinces and cities in the western region can also achieve high-quality development by implementing high-quality development paths. However, considering the differences in resource endowments and economic levels among provinces and cities, when choosing differentiated paths for high-quality development of the construction industry, it is necessary to adapt to local conditions, while also avoiding making choices based on regional divisions in the east, middle, and west. Priority should be given to choosing development paths that are highly applicable and meet one’s own development conditions.

### Difficulties in implementing NCA and fsQCA combination methods

The fuzzy set qualitative comparison method is a fuzzy set method based on fuzzy mathematics, which can quickly analyze complex systems and social phenomena, explore the essential laws of phenomena, and propose effective solutions^49^. Combining system theory and system analysis, mathematical models can be used to describe. Its mathematical model includes defining elements, evaluating element types, and describing element sufficiency. To solve the linear programming problem of mathematical model formulation, existing software including “MANAGER”, “Simplex Method” and “CAE (Computer Aided Engineering)” developed in the UK was also used, among which “MANAGER” is the most convenient to use and has the fastest calculation speed. Compared with previous fuzzy set methods, the fsQCA method can optimize the system by using the objective function or the vector of the entire function, thereby solving the problem of environmental impact.

In the construction industry, a combination of NCA and fsQCA^60^ methods is used for research, and the implementation difficulties mainly include the following two aspects. Firstly, high-quality development is influenced by various factors, and the relevant data of these influencing factors are all from different websites or statistical yearbooks, which increases the difficulty of data collection. Secondly, it is necessary to process the collected data to better utilize NCA and fsQCA for analysis, which is the most complex and difficult process. For example, in the necessity analysis process, if the utility and bottleneck level analysis results of the NCA method are different from the fsQCA necessity test analysis results, it is necessary to adjust the conditional variables, and this process requires attempting to replace multiple conditional variables and performing multiple operations to obtain consistent results, which requires a lot of work to complete.

### Opportunities and challenges for high-quality development of the construction industry

The high-quality development of the construction industry helps to quickly respond to global initiatives for carbon neutrality and achieve a greener future, which is an opportunity. However, there are also many challenges in the formulation, application, and implementation of regulations, materials, technology, and financing mechanisms. Although this article analyzes the main influencing factors of the five development dimensions, it is still necessary to establish a legal system for low-carbon development to provide research support, establish low-carbon standards to regulate market behavior, establish subsidy mechanisms and mandatory systems to guide and ensure steady low-carbon development. We also need to accelerate research and development and expand the proportion of low-carbon material applications, prioritizing the use of low-carbon materials such as low-carbon concrete, ecological bricks, and ecological cement. In terms of technology, it is necessary to vigorously promote BIM-based prefabricated construction technology, intelligent construction technology, etc. In addition, the government and enterprises need to hold regular consultations, build a communication platform between government, banks, and enterprises, establish a green credit channel, and optimize the approval process and other financing mechanisms to ensure the financing of construction enterprises.

### Theoretical contributions

Firstly, unlike previous studies that have mostly explored industrial development issues from a macro perspective, high-quality development in the construction industry is a complex and systematic process. Although macro or industry data can be used to grasp trends and propose relevant theoretical paths, there is a lack of practical application of theoretical paths and analysis of their usability. This article takes construction industry data as a sample and analyzes it from multiple dimensions of high-quality development, proposing practical and feasible paths and their different applicability.

Secondly, it provides new ideas and solutions to solve the problem of high carbon emissions in the construction industry. This article analyzes and argues from the perspective of configuration from multiple dimensions of high-quality development in the construction industry, providing a convincing explanation: the coupling of configuration effects among different influencing factors has an impact on the development of the construction industry, and it also clarifies the multiple implementation paths for high-quality development in the construction industry. Its experience can also provide a reference for the high-quality development of the construction industry in other countries.

Finally, the NCA method is used as a supplement to the QCA method. Based on the necessary causal relationship, NCA analysis reveals that any single condition does not constitute a limiting factor for the development of the construction industry. Different combinations of elements can achieve high-quality development of the construction industry, that is, there are equivalent adaptive paths for the development of the construction industry in different regions under different scenarios. The applicability of these paths is further studied in combination with the characteristics of the paths and regions. The inspiration for optimizing the high-quality development environment of the construction industry is that although there are differences in innovation, coordination, green, open, and shared development among provinces and cities, this does not necessarily hinder these regions from promoting the development of the construction industry through different configuration paths. In addition, by studying the applicability of the path, the research conclusion can find the most suitable and efficient development path for non-high-quality development areas. These research conclusions also provide empirical evidence for exploring differentiated paths for high-quality development of the construction industry in various provinces and cities, and provide reference for improving the efficiency of high-quality development of the construction industry.

## Conclusion and remarks

### Conclusion

1. The level of scientific and technological innovation, the degree of structuring, and the scale of prefabricated buildings are the core conditions, and any single factor among the five factors of the development of the construction industry, such as the level of scientific and technological innovation, the degree of structuring, the scale of prefabricated buildings, the vitality of the external market and resources and environment, do not constitute the necessary conditions.
2. There are three high-quality development paths for the construction industry, namely the open sharing development path, the open innovation coordinated development path, and the linkage development path led by the open innovation coordination. The last configuration path contains the most representative cases and has strong applicability.
3. There are two paths to produce non-high-quality development, and they are not simply opposite to the path to produce high-quality development, which reflects the asymmetry of factors and results of the development of the construction industry.

### Revelation

First and foremost, China’s provincial governments (autonomous region, municipal) would have to give full play to their guiding role, attach importance to the development of scientific and technological innovation, and endeavor to improve the standard of scientific and technological innovation; improve the structure of the construction industry, the government should strengthen strategic cooperation with large and medium-sized central construction firms or with state-owned construction firms; Vigorously developing prefabricated buildings, expanding the number of industrial bases and expanding the scale of prefabricated buildings; Strongly integrating the “Belt and Road” strategy, opening up overseas engineering markets, enhancing the vitality of overseas markets and optimizing the existing resource environment to promote high quality and positive development of the building industry.

Secondly, focus on the linkage and matching of multiple factors. A single factor cannot meet the high-quality development needs of the construction industry, and attention should be paid to the coupling and linkage matching between multiple factors. The open-sharing development path shows that breaking through the constraints of a lack of technological innovation level and prefabricated building scale, enhancing the vitality of the external market, and optimizing resource and environmental conditions may be an effective way to promote high-quality development of the construction industry.

Finally, the economic level and resource endowment of each province (district, city) vary, and a development path with strong applicability should be chosen according to local conditions. While at the same time combining its conditions and regional differences and referring to the case characteristics, optimizing the combination of various factors to improve the level of development of the construction industry.

## Data Availability

Publicly available datasets were analyzed in this study. This data can be found here (1) the rate of technical equipment, the number of employees in the construction industry, and the whole society come from the China Statistical Yearbook. https://www.stats.gov.cn. (2) the output value data of the construction industry comes from the China Statistical Yearbook of the construction industry, the assembled industrial base data comes from the website of the Ministry of Housing and Construction, and the turnover data of foreign contracted projects comes from the website of the Ministry of Commerce. http://www.mofcom.gov.cn.
